# Physiologic Determinants of Exercise Capacity in Pulmonary Langerhans Cell Histiocytosis: A Multidimensional Analysis

**DOI:** 10.1371/journal.pone.0170035

**Published:** 2017-01-10

**Authors:** Camille Rolland-Debord, Stephanie Fry, Jonathan Giovannelli, Carole Langlois, Nicolas Bricout, Bernard Aguilaniu, Agnes Bellocq, Olivier Le Rouzic, Stephane Dominique, Alain Delobbe, Geraldine François, Abdellatif Tazi, Benoit Wallaert, Cecile Chenivesse

**Affiliations:** 1 Service de Pneumologie et ImmunoAllergologie, Centre de Compétence des Maladies Pulmonaires Rares, CHU Lille, Univ. Lille, Lille, France; 2 Service de Pneumologie et Réanimation médicale, APHP-Groupe Hospitalier Pitié-Salpêtrière Charles Foix, Paris, France; 3 UMRS 1158 Neurophysiologie respiratoire expérimentale et clinique, Sorbonne Universités, UPMC Univ Paris 06, INSERM, Paris, France; 4 Pôle de Santé Publique, Pharmacie et Pharmacologie, CHU Lille, Univ. Lille, Lille, France; 5 Service de Biostatistiques, CHU Lille, Univ. Lille, Lille, France; 6 Service de Radiologie, CHU Lille, Univ. Lille, Lille, France; 7 Université Joseph-Fourrier, Faculté de médecine, La Tronche, France; 8 Service d’Explorations Fonctionnelles de la Respiration, de l’Exercice et de la Dyspnée, APHP-Groupe Hospitalier Pitié-Salpêtrière Charles Foix, Paris, France; 9 Clinique Pneumologique, Hôpital Charles Nicolle, Université de Rouen, Centre Hospitalier Universitaire de Rouen, Rouen, France; 10 Service de Pneumologie, Centre Hospitalier Régional de la citadelle, Liège, Belgique; 11 Service de Pneumologie et Réanimation, Centre Hospitalier Universitaire d’Amiens, Université de Picardie Jules Verne, Amiens, France; 12 Service de Pneumologie, Centre de Référence de l’Histiocytose Langerhansienne, APHP - Hôpital Saint-Louis, Université Paris-Diderot, Sorbonne Paris Cité; INSERM UMR 1153 CRESS, Equipe de Recherche en Biostatistiques et Epidémiologie Clinique, Paris, France; National and Kapodistrian University of Athens, GREECE

## Abstract

**Background:**

Reduced exercise capacity severely impacts quality of life in pulmonary Langerhans cell histiocytosis. Ascertaining mechanisms that impair exercise capacity is necessary to identify targets for symptomatic treatments.

**Methods:**

Dyspnea, pulmonary function tests and cardiopulmonary exercise test were analysed in 62 study participants. Data were compared between subjects with impaired and normal aerobic capacity (V’O_2_ peak less than 84% versus 84% predicted or more). Data were reduced using a principal component analysis. Multivariate analysis included V’O_2_ peak as the dependent variable and principal components as covariates.

**Results:**

V’O2 peak was reduced in 44 subjects (71%). Subjects with impaired aerobic capacity presented: (i) decreased FEV_1_, FVC, FEV_1_/FVC, DL_CO_ and DL_CO_/VA and increased AaDO_2_, (ii) increased ventilatory equivalents at ventilatory threshold, V_D_/V_T_ peak, AaDO_2_ peak and PaCO_2_ peak and decreased ventilatory reserve and PaO_2_ peak. There was no difference between groups in dyspnea scores. Principal component analysis extracted 4 principal components interpreted as follows: PC1: gas exchange; PC2: “pseudorestriction”; PC3: exercise-induced hyperpnea; PC4: air trapping. Multivariate analysis explained 65% of V’O2 peak. The 4 principal components were independently associated with V’O2 peak (βcoefficients: PC1: 9.3 [4.6; 14], PC2: 7.5 [3; 11.9], PC3: -5.3 [-9.6;-1.], PC4: -9.8 [-14,9;-4.7]).

**Conclusion:**

Impaired exercise capacity is frequent in pulmonary Langerhans cell histiocytosis. It is mainly caused by pulmonary changes but is not associated with increased dyspnea intensity. Therefore, treating the lung represents a relevant approach for improving exercise capacity, even in patients experiencing mild dyspnea.

## Introduction

Langerhans cell histiocytosis is a rare systemic disease characterized by proliferation of Langerhans cells that infiltrate the tissues. In adults, this disorder affects a limited number of organs, predominantly the lungs, skin and bone [1]. Pulmonary Langerhans cell histiocytosis (PLCH) is mostly observed in genetically predisposed young adult smokers [2]. The most common symptoms are cough, breathlessness and fatigue. Chest computed tomography (CT) scan typically shows bilateral micronodular interstitial syndrome, excavated nodules and cysts predominantly located in the upper and middle areas of the lung [3]. Pulmonary hypertension is present in 10% of cases [3] due to infiltration of pulmonary capillaries by Langerhans cells [4] or complicating the interstitial lung disease. Pulmonary function tests (PFT) show various abnormalities including reduction of the diffusing capacity of the lung for carbon monoxide (DL_CO_), decreased vital capacity (VC), increased residual volume (RV) and airway obstruction [5].

PLCH impairs daily life activities and deteriorates quality of life [6,7]. Accordantly, it is associated with decreased maximum oxygen consumption (V’O_2_ peak) and reduced distance walked during a 6-minute walk test [8,9]. The pathophysiologic mechanisms that limit exercise capacity have been poorly investigated and consequently, the effect of symptomatic treatments is unknown. One study reported correlations between V’O_2_ peak and the resting dead space/tidal volume ratio (V_D_/V_T_), RV, resting alveolar-to-arterial oxygen tension difference (AaDO_2_) and DL_CO_ [8]. However, in that study the relationship between V’O_2_ peak and variables at exercise, which are rather impaired and provide a better understanding of exercise capacity alteration, were not studied.

Cardiopulmonary exercise testing (CPET) is a crucial tool for determining the respective roles of cardiovascular, respiratory or peripheral responses in exercise limitation [10]. For instance, in patients with sarcoidosis, CPET revealed that exercise capacity is essentially limited by the cardiocirculatory response in mild-to-moderate stages of the disease, while it is mainly limited by gas exchange impairment in severe stages [11]. Moreover, CPET can reveal exercise-induced physiologic dysfunction able to explain impaired exercise capacity in patients with normal PFT [10]. In PLCH, AaDO_2_ at peak exercise can be increased whereas DL_CO_ is normal [8]. However, no data are available on the relationship between V’O_2_ peak and physiologic variables measured during exercise. Hence, this study was designed to investigate the physiologic determinants of reduced exercise capacity in patients with PLCH.

## Methods

### Design

We conducted a multicenter retrospective study in 8 hospitals. All patients referred between 1993 and 2013 at the time of diagnosis or during follow-up of PLCH were screened for inclusion. Patients who had performed several CPET were included only once. In this condition, only the first available CPET was selected. Inclusion criteria were: (i) a diagnosis of PLCH confirmed by histologic examination or a combination of typical clinical history and chest CT scan features, (ii) a maximal CPET including arterial blood gases at peak exercise.

### Ethics

The study was approved by the Institutional Review Board of the French Learned Society for Pulmonology (CEPRO 2012 039).

### Dyspnea score

Limitation of activity related to dyspnea was evaluated using the modified Medical Research Council (mMRC) scale.

### Chest CT scans

Chest CT scans performed up to six months after CPET were analysed. Chest CT scans performed more than 6 months after CPET were not considered for analysis. The prevalence of nodular and cystic abnormalities was analysed by two blinded radiologists (K.Y. and N.B.). Chest CT changes were classified as previously described [12]. The extent of nodular lesions was categorised as follows: no nodules, mild, moderate, or diffuse nodules. The extent of cystic lesions was classified by the percentages of the lung surface analysed: no cyst, <25%, 25–49%, 50–75%, or >75%.

### Echocardiography

Echocardiography performed up to 6 months after CPET were analysed. Pulmonary artery pressure was collected.

### Pulmonary function tests

Forced vital capacity (FVC), forced expiratory volume in 1 s (FEV_1_), FEV_1_ to FVC ratio, total lung capacity (TLC), functional residual capacity (FRC) and RV were evaluated by plethysmography. DLco was measured using the single-breath method after adjusting for hemoglobin concentration and according to Cotes’ equation. Predicted normal values were derived from standard equations [13]. Values were expressed as percentages of the predicted normal values calculated according to gender, weight, and age. Reference equations were taken from ERS [14].

### CardioPulmonary Exercise Testing

CPET was performed according to a similar standardized protocol in the 8 centers on the same day as PFT. Short and long acting bronchodilators were withheld for at least 8 and 24 hours before the exam, respectively. The CPET protocol consisted in a symptom-limited incremental exercise test on an ergometric bicycle including a warm-up period of 3 min at 20 W followed by a progressively increasing work rate in a ramp fashion (8 to 30 W/min) and then 3 min recovery. It is described in details in previous publications [15]. Immediately after exercise, subjects were asked to score breathlessness and muscle fatigue at the end of exercise using Borg scales. Limitation of aerobic capacity was defined as V’O_2_ peak < 84% of predicted [10].

### Statistical analyses

Continuous variables were expressed as mean ± standard deviation or median and interquartile range according to their normal or non-normal distribution. Qualitative variables were expressed as sample size and percentage of the population. Continuous variables were compared between groups using a Student’s T test or a Mann-Whitney test. Qualitative variables were compared using Chi-square or Fischer’s test. Correlation between V’O_2_ peak, CPET and PFT variables were evaluated using a Pearson’s coefficient or a Spearman’s coefficient. Principal component analysis (PCA) was performed in order to resume the data in principal components. When a variable was expressed both in absolute value and percentage of predicted, only the variable expressed as percentage of predicted was selected. We retained factors with eigenvalue greater than 1. Rotation was performed by the varimax method. Linear regression was performed using V’O_2_ peak as the dependent variable and PCA-extracted factors as covariates. Linear regression was adjusted for age, gender, weight, height, active smoking (yes/no) and duration of disease. The normality of the distribution of residuals and the diagnosis of homoscedasticity were evaluated graphically. Statistical analyses were performed on SPSS (IBM SPSS Statistics 22); the threshold for statistical significance was set to p < 0.05.

## Results

### Sociodemographic and clinical characteristics

A total of 62 subjects (27 men, 35 women) with a mean age of 37 ± 10 years were included. The diagnosis of PLCH was confirmed by suggestive clinical features associated with typical CT lesions in 63% of cases, surgical lung biopsy in 31% of cases and extrathoracic biopsy in 6% of cases. Subjects were Caucasians in 94% of cases. The study population comprised 66% of smokers, 31% of ex-smokers and 3% of non-smokers. The median interval between the date of diagnosis and the date of CPET was 3 years. No statistically significant difference was observed for sociodemographic data and dyspnea severity between subjects with or without reduced V’O_2_ peak (Table 1).

**Table 1:** Sociodemographic and clinical characteristics.

	Total	V’O_2_ peak ≥ 84% pred	V’O_2_ peak < 84% pred	p
**Sociodemographic data**	**n = 62**	**n = 18**	**n = 44**	
Gender, male	27 (43%)	5 (28%)	22 (50%)	0.109
Age, years	37 ± 10	39 ± 10	36 ± 11	0.323
BMI, kg/m^2^	21.8 (6.4)	21.9 (6.8)	21.5 (6.4)	0.625
Ethnic group, Caucasian	52 (94%)	16 (89%)	36 (82%)	0.254
History of Smoking				
Smoker	41 (66%)	13 (72%)	28 (64%)	0.598
Ex-smoker	19 (31%)	5 (28%)	14 (32%)	
Non-smoker	2 (3%)	0 (0%)	2 (4.5%)	
**Clinical data**	**n = 62**	**n = 18**	**n = 44**	
Interval between diagnosis and evaluation, years	3 (6)	1 (6)	4 (7)	0.262
mMRC scale	1 (1)	0.8 (1)	0.9 (2)	0.8
**CT scan data**	**n = 37**	**13**	**24**	
Nodular score	6 (3.5–11)	6 (3.5–9)	7 (3.25–14)	0.59
Low	20 (54%)	8 (62%)	12 (50%)	
Intermediate	9 (24%)	4 (31%)	5 (21%)	
High	8 (22%)	1 (7%)	7 (29%)	
Cystic score	7 (6–13.5)	6 (5–6)	9.5 (6–14)	0.024
Low	19 (51%)	11 (85%)	8 (33%)	
Intermediate	9 (24%)	0 (0%)	9 (37%)	
High	5 (14%)	2 (15%)	3 (13%)	
Very high	4 (11%)	0 (0%)	4 (17%)	

BMI: body mass index, CT: computed tomography, mMRC: modified Medical Research Council, V’O_2_ peak: maximal oxygen consumption. Results are expressed as n(%), mean ± SD or median (Q1-Q3)

### CT scan

A CT scan was available in 37 cases (Table 1). CT scans revealed varying degrees of nodular and cystic abnormalities: 30/37 patients had both nodules and cysts; 3 patients had an isolated cystic pattern, and 4 patients had an isolated nodular pattern. The mean CT nodular score was 7.7 ± 5.6 and the mean CT cystic score was 9.1 ± 6.3. The mean cystic score was statistically higher in subjects with reduced V’O_2_ peak.

### Echocardiography

An echocardiography was available in 21 cases (median sPAP 29 mmHg (23–37)). Of those, 14 had reduced V’O_2_ peak and 7 had normal V’O_2_ peak (sPAP 28 (22–37) versus 29 (28–37); p = 0.65). Pulmonary hypertension was suspected (sPAPs > 35mmHg) in 6 patients. No right catheterization was available. No correlation was observed between sPAP and V’O_2_ peak; r = 0.14, p = 0.54).

### Pulmonary function tests

PFT results are summarised in Table 2. In subjects with reduced V’O_2_ peak, FEV_1_, FVC, FEV_1_/FVC, DL_CO_ and DL_CO_/VA were significantly lower and AaDO_2_ was significantly higher compared with subjects with normal V’O_2_ peak. No statistically significant difference was observed between groups in RV, FRC, TLC, RV/TLC, PaO_2_ and PaCO_2_.

**Table 2:** Pulmonary function tests and incremental cycle exercise results.

	Total	V’O_2_ peak ≥ 84% pred	V’O_2_ peak < 84% pred	p
**Pulmonary Function Test**	**n = 62**	**n = 18**	**n = 44**	
FEV_1_, % pred	74 ± 25	95 ± 16	66 ± 23	< 0.001
FVC, % pred	91 ± 22	106 ± 17	84 ± 20	< 0.001
FEV_1_/FVC, %	68 ± 14	75 ± 7	65 ± 15	< 0.001
RV, % pred	129 ± 50	117 ± 25	115 (81)	0.821
FRC, % pred	115 ± 30	119 ± 23	113 ± 32	0.508
TLC, % pred	102 ± 19	108 ± 14	99 ± 21	0.112
RV/TLC, %	36 (17)	33 ± 8	37 (20)	0.352
DL_CO_, % pred	61 ± 19	75 ± 19	55 ± 15	< 0.001
PaO_2_, mmHg	87 ± 13	91 ± 15	86 ± 11	0.118
PaCO_2_, mmHg	37 ± 4	37 ± 5	37 ± 4	0.656
AaDO_2_, mmHg	18 (23)	10 (15)	22 ± 13	0.049
**Incremental cycle exercise**	**n = 62**	**n = 18**	**n = 44**	
Workload peak, % pred	71 ± 19	85 ± 16	65 ± 17	< 0.001
V’O_2_ peak, % pred	73.7 ± 17.9	95 ± 9	66 (15)	< 0.001
V’E peak, L/min	67 ± 22	72 ± 14	65 ± 25	0.181
V’E/V’CO_2_ peak	38 ± 8	36 ± 7	38 (12)	0.113
V’E/V’O_2_ VT	33 (12)	30 (6)	36 ± 7	0.023
V’E/V’CO_2_ VT	34 (12)	33 ± 7	38 ± 8	0.047
BR, %	31 (28)	37 ± 12	25 ± 21	0.005
V_D_/V_T_ peak	0.33 ± 0.11	0.27 ± 0.11	0.36 ± 0.11	0.006
V_D_/V_T_ rest	0.38 ± 0.13	0.35 ± 0.09	0.39 ± 0.14	0.337
PaO_2_ peak, mmHg	79 ± 18	94 (24)	74 ± 17	0.001
PaCO_2_ peak, mmHg	38 ± 5	35 ± 4	39 ± 5	0.005
AaDO_2_ peak, mmHg	35 (26)	22 (11)	38 ± 15	0.004

AaDO_2_: Alveolar-to-arterial oxygen tension difference; BR: Breathing Reserve; DL_CO_: Diffusing Capacity of the Lung for carbon monoxide; FEV_1_: Forced Expiratory Volume in 1 s; FRC: Functional Residual Capacity; FVC: Forced Vital Capacity; HR: Heart Rate; PaCO2: partial pressure of carbon dioxide in arterial blood; PaO_2_: Partial pressure of oxygen in arterial blood; RR: Respiratory Rate; RV: Residual Volume; TLC: Total Lung Capacity; VA: Alveolar Volume; V_D_/ V_T_: dead space/tidal volume ratio; V’E: minute ventilation; V’E/V’O_2_: ventilatory equivalent for O_2_; V’E/V’CO_2_: ventilatory equivalent for CO_2_; V_T_: Tidal Volume; VT: ventilatory threshold; V’O_2_: oxygen consumption.

Results are expressed as mean ± SD or median (Q1-Q3)

### CardioPulmonary Exercise Testing

A maximal symptom-limited exercise was obtained in all cases judged on symptoms and/or a functional limitation as all patients had at least one of the following: breathing reserve (BR) < 15%, peak heart rate (HR) > 90% of predicted, peak lactate > 7 mEq/L, peak PaO2 < 55 mmHg and peak ventilator equivalent for O_2_ (VE/VO2) > 35 or peak Respiratory Exchange Ratio > 1.15.(10) Subjects attributed discontinuation of exercise to breathlessness in 1/3 of cases (n = 14), muscle fatigue in 1/3 of cases (n = 12) and both in 1/3 of cases (n = 17). The CPET results are summarized in Table 2. The median dyspnoea Borg intensity at the end of maximum incremental exercise was scored as severe with no statistically significant difference between the 2 groups (5(7) versus 5.5(4), p = 0.74). V’O_2_ peak was reduced in 44 (71%) subjects. No HR, blood pressure or electrocardiographic abnormalities were observed. Oxygen pulse was greater than 70% of predicted in 54 of the 62 subjects and displayed normal kinetics in all subjects. It is of note that 6 of the 8 subjects with a lower than 70% predicted oxygen pulse had performed echocardiography showing no sign of pulmonary hypertension (pulmonary artery systolic pressure lower than 40 mmHg) or left ventricular dysfunction (data not shown). Subjects with decreased V’O_2_ peak had significantly higher ventilatory equivalents at ventilator threshold (VT), V_D_/V_T_ peak, AaDO_2_ peak and PaCO_2_ peak and lower BR, PaO_2_ peak and oxygen pulse. In 21 patients with a recent echocardiography, pulmonary artery pressure did not correlate with VD/VT at rest and peak (data not shown).

### Correlations between V’O2 peak and CPET/PFT variables

V’O_2_ peak was not correlated with either mMRC score (r = -0.21, p = 0.11) or Borg score (r = 0.023; p = 0.89). Nineteen of the 40 PFT and CPET variables recorded were statistically correlated with V’O_2_ peak (Table 3). We did not consider the correlation between VO_2_ peak and pulse oxygen, which is evidently high but not informative, as the calculation of pulse oxygen includes VO_2_ peak.

**Table 3:** Variables statistically correlated with V’O_2_ peak.

	n	r	p
FEV_1_, % pred	62	0.709	< 0.001
FVC, % pred	62	0.543	< 0.001
FEV_1_/FVC, %	62	0.582	< 0.001
RV, % pred	57	-0.286	0.031
RV/TLC, %	58	-0.291	0.027
DL_CO_, % pred	57	0.662	< 0.001
DL_CO_/VA, % pred	53	0.533	< 0.001
PaO_2_ rest, mmHg	59	0.409	0.001
AaDO_2_ rest, mmHg	54	-0.484	< 0.001
V’E peak, L/min	62	0.349	0.005
V’E/V’CO_2_ peak	62	-0.297	0.019
V’E/V’O_2_ VT	58	-0.436	0.001
V’E/V’CO_2_ VT	58	-0.390	0.002
BR, %	62	0.410	0.001
V_D_/V_T_ peak	58	-0.475	< 0.001
V_D_/V_T_ rest	59	-0.265	0.043
PaO_2_ peak, mmHg	58	0.553	< 0.001
PaCO_2_ peak, mmHg	58	-0.445	< 0.001
AaDO_2_ peak, mmHg	54	-0.504	< 0.001

AaDO_2_: alveolar-to-arterial oxygen tension difference; BR: breathing reserve; DL_CO_: Diffusing capacity of the lung for carbon monoxide; FEV_1_: forced expiratory volume in 1s; FRC: functional residual capacity; FVC: Forced vital capacity; PaCO_2_: partial pressure of carbon dioxide in arterial blood; PaO_2_: partial pressure of oxygen in arterial blood; RV: residual volume; TLC: total lung capacity; VA: alveolar volume; V_D_/V_T_: dead space/tidal volume ratio; V’E: minute ventilation; V’E/V’CO_2_: ventilatory equivalent for CO_2_; V’E/V’O_2_: ventilatory equivalent for O_2_; V’O_2_: maximal oxygen consumption

### Principal component analysis

After exclusion of the subjects with any missing data, 45 subjects were included in the principal component analysis. Subjects with and without missing data showed similar sociodemographic, PFT and CPET characteristics apart from higher AaDO_2_ at rest and peak and lower PaO_2_ peak in the missing data group (S1 Table).

Four factors had eigenvalues greater than 1. The first factor alone explained 48% of total variance. The 4 factors explained 79% of total variance. The component matrix after rotation is shown in Table 4.

**Table 4:** Correlations between the four principal components and CPET and PFT variables, n = 45.

	Principal component			
	1	2	3	4
AaDO_2_ peak, mmHg	-0.804	-0.354	0.223	-0.108
AaDO_2_ rest, mmHg	-0.790	0.023	0.317	0.272
PaO_2_ rest, mmHg	0.775	0.100	-0.150	-0.267
PaO_2_ peak, mmHg	0.743	0.566	-0.081	-0.054
DLco/VA, % pred	0.683	0.188	-0.398	0.053
FEV_1_/FVC, %	0.551	0.354	-0.058	-0.493
FVC, %	-0.015	0.872	-0.127	-0.176
FEV_1_, %	0.302	0.803	-0.118	-0.420
PaCO_2_ peak, mmHg	-0.140	-0.735	-0.506	0.131
V_D_/V_T_ peak	-0.387	-0.619	0.495	0.143
BR, %	0.232	0.595	-0.584	-0.082
V_D_/ V_T_, resting	-0.396	-0.571	0.210	0.183
DL_CO_, % pred	0.504	0.567	-0.416	-0.058
V’E/V’CO_2_ peak	-0.227	-0.118	0.910	-0.007
V’E/V’O_2_ VT	-0.504	-0.070	0.762	0.243
V’E/V’CO_2_ VT	-0.416	-0.138	0.726	0.323
RV/TLC, %	-0.071	-0.316	0.312	0.845
RV, % pred	0.017	-0.110	0.229	0.818
V’E peak, L/min	0.253	0.131	0.283	-0.718

AaDO_2_: Alveolar-to-arterial Oxygen tension Difference; BR: Breathing Reserve; DL_CO_: Diffusing capacity of the Lung for Carbon monoxide; FEV_1_: Forced Expiratory Volume in 1 s; FVC: Forced Vital Capacity; PaCO_2_: Partial pressure of carbon dioxide in arterial blood; PaO_2_: Partial pressure of oxygen in arterial blood; RV: Residual Volume; TLC: Total Lung Capacity; VA: Alveolar Volume; V_D_/V_T_: dead space/tidal volume ratio; V’E: minute ventilation; V’E/V’CO_2_: ventilatory equivalent for CO_2_; V’E/V’O_2_: ventilatory equivalent for O_2_

Principal component 1 (PC1) was associated with variables measuring gas exchange, as it was negatively correlated with AaDO_2_ at rest (r = -0.79) and peak (r = -0.804) and positively correlated with PaO_2_ at rest (r = 0.775) and peak (r = 0.743) and DL_CO_/VA (r = 0.683). PC1 was also associated with FEV_1_/FVC (r = 0.551). Principal component 2 (PC2) was associated with lung volumes, as it was positively correlated with FVC (r = 0.872) and FEV_1_ (r = 0.803) and with variables including lung volumes in their calculation: DL_CO_ (r = 0.567), BR (r = 0.595), V_D_/V_T_ peak (r = -0.619) and V_D_/V_T_ at rest (r = -0.571). PC2 was also negatively correlated with PaCO_2_ peak (r = -0.735). Principal component 3 (PC3) was correlated with ventilatory equivalents: peak V’E/V’CO_2_ (r = 0.910), VT V’E/V’CO_2_ (r = 0.726) and VT V’E/V’O_2_ (r = 0.762). Principal component 4 (PC4) was positively correlated with measurements of air trapping including RV (r = 0.818) and RV/TLC (r = 0.845) and negatively correlated with V’E peak (r = -0.718).

### Association between V’O_2_ peak and PFT and CPET variables

V’O_2_ peak was independently correlated with the 4 principal components (Table 5). Overall, 65% of the variability of V’O_2_ peak was explained by the regression model (R^2^ = 0.65). Graphic analysis showed that the residues of linear regression followed a normal distribution and were scattered homogeneously over the spectrum of values of V’O_2_ peak, confirming the homoscedasticity of the model.

**Table 5:** Association between V’O_2_ peak and the four principal components, n = 45.

	Beta coefficient	95% confidence interval	p
PC1	9.3	[4.6;14]	< 0.001
PC2	7.5	[3;11.9]	0.002
PC3	-5.3	[-9.6;-1]	0.017
PC4	-9.8	[-14.9;-4.7]	< 0.001
Age, year	0.6	[0;1.1]	0.047
Gender, male	9.3	[-2.3;20.9]	0.114
Weight, kg	-0.4	[-0.8;0]	0.316
Height, m	21.5	[-55.6;98.7]	0.575
Disease duration, year	0.8	[-0.2;1.7]	0.103
Active smoking, yes	-3	[-13.2;7.2]	0.552

PC: principal component

## Discussion

This study provides a revisited analysis of physiologic determinants of exercise capacity in PLCH including the largest population studied until now and a more comprehensive set of measurements on exercise endorsed by a principal component analysis. The main results of this study can be summarized as follows: (i) impaired exercise capacity is common among patients with moderate PLCH, (ii) it is caused by multifactorial alteration of lung function including alteration of gas exchange, air trapping, pseudorestriction and exercise-induced hyperventilation (iii) it is not associated with increased dyspnea intensity.

Crausman et al. reported that patients with PLCH have severe alteration of aerobic capacity (mean V’O_2_ peak: 44% of predicted) [8]. The authors attributed exercise limitation to pulmonary hypertension based on the strong correlation between V’O_2_ peak and the increased V_D_/V_T_ ratio at rest, which is an indirect but nonspecific sign of pulmonary hypertension. This hypothesis was not corroborated by a direct measurement of pulmonary artery pressure. In our study, the maximum exercise capacity was less severely impaired (mean V’O_2_ peak: 74% of predicted). This may be due a less severe disease as suggested by low dyspnea scores (median mMRC: 1 versus 2 in previous studies [7,12,16] and only slightly altered PFT (mean FVC: 91% and mean DL_CO_: 61%) or a lower prevalence of pulmonary hypertension that was estimated at 29% IC95 [9 to 48%] in a subgroup of 21 with echocardiography measurements in our study. Our results show that the reduction of aerobic capacity in PLCH has a multifactorial origin, involving alteration of gas exchange, air trapping, “pseudorestriction” and exercise-induced hyperpnea. It is of note that although exercise was limited by pulmonary changes there was no difference in dyspnea intensity between patients with reduced and normal exercise capacity. This advocates that respiratory symptoms may not be necessarily warming sign for lung related reduction of exercise capacity in PLCH.

In our study, the alteration of gas exchange (PC1) was the main determinant of exercise capacity. All variables measuring gas exchange (DL_CO_, DL_CO_/VA and AaDO_2_) were more severely altered in patients with decreased aerobic capacity and were correlated with PC1 that was strongly associated with V’O_2_ peak. In PLCH, the alteration of gas exchange is mainly due to cystic destruction of lung parenchyma. The correlation between FEV_1_/FVC and PC1 is likely due to the strong relationship between bronchial obstruction and the extent of cysts as reported previously [12,16] and also found in our study in the sub-group of 37 patients having a CT scan (data not shown).

Air trapping (PC4) was the second major factor responsible for limitation of exercise capacity. In PLCH, air trapping is caused by obstruction of distal airways due to several mechanisms including infiltration by histiocytes and compression of airways by cysts. RV is increased very early in PLCH(8) and then remains nearly unchanged during the course of the disease [7]. This may explain the absence of any statistically significant difference in RV between patients with and without impaired aerobic capacity. In chronic obstructive pulmonary disease, air trapping impacts exercise capacity by inducing dynamic hyperinflation [17, 18] leading to reduced inspiratory capacity and finally to an inability to increase V_T_ [19]. We did not have any flow-volume loops available to confirm dynamic hyperinflation. However, the correlation between V’E and PC4 is an indirect argument in favour of the development of dynamic hyperinflation, as hyperventilation induces dynamic hyperinflation in obstructive diseases [20].

The third factor limiting exercise in our study was ventilatory abnormalities (PC2). Dynamic volumes (FEV_1_, FVC) were decreased in patients with impaired aerobic capacity, while static volumes (TLC, FRC) were similar in patients with and without impaired aerobic capacity. This functional profile—commonly called “pseudorestriction”—is typically observed in bronchiolar diseases, in which decreased pulmonary compliance due to cellular infiltration of the lung decreases dynamic volumes, while air trapping increases RV leading to globally normal static volumes [21]. Decreased FEV_1_ and FVC were unlikely due to airway obstruction, as PC2 did not correlate with FEV_1_/FVC. Dynamic volumes were inversely correlated with PaCO_2_ reflecting alveolar hypoventilation during exercise. They were also inversely correlated with V_D_/V_T_. Although we can not rule out the participation of a vascular dysfunction, V_D_/V_T_ was correlated with “hypoventilation-related” (PC2) variables and not with “vascular-related” variables such as hyperventilation. Furthermore, the absence of severe clinical and functional impairment, the normality of cardiovascular adaptation during exercise and the absence of correlation of systolic pulmonary artery hypertension with V_D_/V_T_ and V’O_2_ peak [22] on available echocardiography make the hypothesis of a relationship between pulmonary hypertension and aerobic capacity impairment unlikely [9].

Finally, our study showed that hyperpnea (PC3) independently limited exercise. Increased ventilation was not associated with hypocapnia and thus was appropriate to metabolic requests.

With the exception of specific treatment of pulmonary hypertension, when appropriate, [9,23] no drug treatment has been shown to improve exercise capacity in PLCH. The impact of smoking cessation, systemic steroids or cladribine on exercise capacity has not been evaluated. Pulmonary rehabilitation, which has been shown to improve exercise capacity in several interstitial lung diseases, has not been specifically evaluated in PLCH. Based on the results of the present study, the benefit of pulmonary symptomatic treatments on exercise capacity can be discussed. In non cystic diffuse interstitial lung disease, oxygen supplementation has been showed to improve V’O_2_ peak [24] and the six-minute walking test distance [25] in patients with gas exchange impairment. However, the benefit of portable oxygen therapy on physical activities of daily living remains unknown. Bronchodilators decrease dynamic hyperinflation and improve exercise capacity in chronic obstructive pulmonary disease, even when airway obstruction is mild [26]. However, the effect of bronchodilators in PLCH, in which the mechanisms of airway obstruction are partly different from those involved in chronic obstructive pulmonary disease, has not been evaluated.

### Strengths and limitations

This study provides new data on exercise capacity in the largest population of PLCH patients published to date. The characteristics of the study population were similar to those reported in the literature, with an equivalent proportion of men and woman, a mean age in the range 20–40 years, predominantly Caucasian ethnic origin and a history of smoking in more than 90% of patients [3,6,7]. Biases related to the retrospective design of the study were limited by standardised recording of objective measurements and by systematic review of all CPET based on international guidelines [10] by a single investigator. However, 17 subjects showing more severe gaz exchange impairment could not be included in the multivariate analysis. Thus, we must keep in mind that our results concern mild to moderate stages of PLCH. CT scan were available in only half of the population preventing from inclusion of CT scan changes in the multivariate analysis. This is due to the fact that we only collected CT scans performed close in time to PFT and CPET in the aim to have relevant relationships between CT scan features and functional data. Indeed, PLCH radiological lesions progress over time [27]. For similar reasons, sPAP coud not be included in the multivariate analysis. However, among the 21 patients having an echocardiography no correlation was observed between sPAP and V’O_2_ peak. If we extrapolate these results to the whole population, which seems to have similar sociodemographic, PFT and CPET characteristics (S2 Table), sPAP should not be integrated in the multivariate analysis.

## Conclusion

In conclusion, exercise limitation is frequent in PLCH and is mainly caused by multifactorial pulmonary changes including alteration of gas exchange, air trapping, “pseudorestriction” and hyperpnea but does not appear to be associated with increased dyspnea. This suggests that treating the lung represents an approach for improving exercise, even in patients experiencing mild dyspnea. In particular, our results suggest assessing the effect of therapies targeting air trapping such as bronchodilators on aerobic capacity.

## Supporting Information

S1 Table Comparison of sociodemographic, PFT and CPET data between subjects with and without missing data. (DOCX)

S2 Table Comparison of sociodemographic, PFT and CPET data between subjects with and without echocardiography. (DOCX)

S3 Table Data set as a Supporting Information file. (XLSX)
