# Supplementary material for: Physiologic Determinants of Exercise Capacity in Pulmonary Langerhans Cell Histiocytosis: A Multidimensional Analysis
Source: PLoS One. 2017 Jan 10;12(1):e0170035. doi: 10.1371/journal.pone.0170035 (PMC5225005; doi:10.1371/journal.pone.0170035)
Supplement: S1 Table — (DOCX) [file pone.0170035.s001.docx]

|  | **Total** | **Missing data**  **group** | **No missing data**  **group** | **p** |
| --- | --- | --- | --- | --- |
| **Sociodemographic data** | **n=62** | **n= 17** | **n=45** |  |
| Gender, male | 27 (43%) | 6 (35%) | 21 (46%) | 0.42 |
| Age, years | 37 ± 10 | 39 ± 10 | 36 ± 10 | 0.36 |
| BMI, kg/m^2^ | 21.8 (6.4) | 22.2 (9.2) | 21.8 (6.1) | 0.91 |
| Ethnic group, Caucasian | 52 (94%) | 12 (75%) | 40 (95%) | 0.08 |
| History of Smoking |  |  |  | 0.06 |
| Smoker | 41 (66%) | 9 (53%) | 32 (71%) |  |
| Ex-smoker | 19 (31%) | 6 (35%) | 13 (29%) |  |
| Non-smoker | 2 (3%) | 2 (12%) | 0 (0%) |  |
| **Clinical data** |  |  |  |  |
| Interval between diagnosis and evaluation, years | 3 (6) | 3 (7) | 4(6) | 0.79 |
| mMRC scale | 1 (1) | 1 (1) | 1 (1) | 0.84 |
| **Pulmonary Function Test** |  |  |  |  |
| FEV_1_, % pred | 74 ± 25 | 65 ± 26 | 78 ± 24 | 0.09 |
| FVC, % pred | 91 ± 22 | 83 ± 22 | 93 ± 21 | 0.12 |
| FEV_1_/FVC, % | 68 ± 14 | 64 ± 15 | 70 ± 13 | 0.08 |
| RV, % pred | 129 ± 50 | 129 ± 55  **(n=12)** | 128 ± 49 | 0.79 |
| FRC, % pred | 115 ± 30 | 102 ± 28  **(n=12)** | 118 ± 29 | 0.13 |
| TLC, % pred | 102 ± 19 | 102 ± 25  **(n=14)** | 102 ± 17 | 0.88 |
| RV/TLC, % | 36 (17) | 33 (24) | 35 (15) | 0.63 |
| DL_CO_, % pred | 61 ± 19 | 57 ± 23  **(n=12)** | 62 ±18 | 0.33 |
| PaO_2_, mmHg | 87 ± 13 | 81 ±11  **(n=14)** | 89 ±13 | 0.08 |
| PaCO_2_, mmHg | 37 ± 4 | 37 ± 5  **(n=14)** | 37 ± 4 | 0.51 |
| AaDO_2_, mmHg | 18 (23) | 33 (24) | 16 (20) | 0.04 |
| **Incremental cycle exercise** |  |  |  |  |
| Workload peak, % pred | 71 ± 19 | 70 ± 21 | 71 ±18 | 0.74 |
| V’O_2_ peak, % pred | 73.7 ± 17.9 | 69 ± 15 | 75.5 ± 18.7 | 0.16 |
| V’E peak, L/min | 67 ± 22 | 60 ± 24 | 69 ± 21 | 0.19 |
| V’E/V’CO_2_ peak | 38 ± 8 | 39 ± 8 | 38 ± 8 | 0.45 |
| V’E/V’O_2_ VT | 33 (12) | 39 (12)  **(n=13)** | 33 (9) | 0.33 |
| V’E/V’CO_2_ VT | 34 (12) | 37 (12) | 34 (11) | 0.61 |
| BR, % | 31 (28) | 26 (33) | 32 (28) | 0.24 |
| V_D_/V_T_ peak | 0.33 ± 0.11 | 0.36 ± 0.10  **(n=14)** | 0.33 ± 0.12 | 0.36 |
| V_D_/V_T_ rest | 0.38 ± 0.13 | 0.36 ± 0.16  **(n=14)** | 0.38 ± 0.12 | 0.94 |
| PaO_2_ peak, mmHg | 79 ± 18 | 68 ± 15  **(n=13)** | 82 ± 16 | 0.002 |
| PaCO_2_ peak, mmHg | 38 ± 5 | 39 ± 5  **(n=13)** | 38 ± 5 | 0.54 |
| AaDO_2_ peak, mmHg | 35 (26) | 51 (9)  **(n=9)** | 33 (24) | 0.003 |
