# Supplementary material for: Physiologic Determinants of Exercise Capacity in Pulmonary Langerhans Cell Histiocytosis: A Multidimensional Analysis
Source: PLoS One. 2017 Jan 10;12(1):e0170035. doi: 10.1371/journal.pone.0170035 (PMC5225005; doi:10.1371/journal.pone.0170035)
Supplement: S2 Table — (DOCX) [file pone.0170035.s002.docx]

|  | **Total** | **Echocardiography**  **group** | **No echocardiography group** | **p** |
| --- | --- | --- | --- | --- |
| **Sociodemographic data** | **n=62** | **n=21** | **n=41** |  |
| Gender, male | 27 (43%) | 11 (52%) | 16 (39%) | 0.31 |
| Age, years | 37 ± 10 | 36 ± 10 | 38 ±11 | 0.78 |
| BMI, kg/m^2^ | 21.8 (6.4) | 23.9 (8.8) | 21.3 (4.5) | 0.17 |
| Ethnic group, Caucasian | 52 (94%) | 17 (94%) | 35 (97%) | 0.61 |
| History of Smoking |  |  |  | 0.09 |
| Smoker | 41 (66%) | 11 (52%) | 30 (73%) |  |
| Ex-smoker | 19 (31%) | 10 (48%) | 9 (22%) |  |
| Non-smoker | 2 (3%) | 0 | 2 (5%) |  |
| **Clinical data** |  |  |  |  |
| Interval between diagnosis and evaluation, years | 3 (6) | 3 (6) | 3 (7) | 0.98 |
| mMRC scale | 1 (1) | 1 (1) | 1 (1) | 0.83 |
| **Pulmonary Function Test** |  |  |  |  |
| FEV_1_, % pred | 74 ± 25 | 77 ± 23 | 73 ± 27 | 0.50 |
| FVC, % pred | 91 ± 22 | 90 ± 23 | 91 ± 22 | 0.94 |
| FEV_1_/FVC, % | 68 ± 14 | 71 ± 12 | 66 ± 15 | 0.32 |
| RV, % pred | 129 ± 50 | 120 ±50 | 132 ± 50 | 0.32 |
| FRC, % pred | 115 ± 30 | 105 ± 21 | 116 ± 30 | 0.17 |
| TLC, % pred | 102 ± 19 | 97 ± 15 | 105 ± 21 | 0.12 |
| RV/TLC, % | 36 (17) | 34 (14) | 38 (17) | 0.44 |
| DL_CO_, % pred | 61 ± 19 | 58 ± 18 | 63 ± 19 | 0.40 |
| PaO_2_, mmHg | 87 ± 13 | 84 ± 23 | 87 ± 12 | 0.88 |
| PaCO_2_, mmHg | 37 ± 4 | 37 ± 4 | 37 ± 5 | 0.99 |
| AaDO_2_, mmHg | 18 (23) | 11 (18) | 18 (24) | 0.41 |
| **Incremental cycle exercise** |  |  |  |  |
| Workload peak, % pred | 71 ± 19 | 69 ± 16 | 72 ± 21 | 0.76 |
| V’O_2_ peak, % pred | 73.7 ± 17.9 | 71.3 ± 19.7 | 75 ± 17 | 0.56 |
| V’E peak, L/min | 67 ± 22 | 70 ± 23 | 65 ± 21 | 0.61 |
| V’E/V’CO_2_ peak | 38 ± 8 | 38 ± 8 | 38 ± 7 | 0.89 |
| V’E/V’O_2_ VT | 33 (12) | 32 (8) | 34 (13) | 0.27 |
| V’E/V’CO_2_ VT | 34 (12) | 33 (8) | 37 (14) | 0.46 |
| BR, % | 31 (28) | 35 (22) | 28 (11) | 0.44 |
| V_D_/V_T_ peak | 0.33 ± 0.11 | 0.35 ± 0.09 | 0.32 ± 0.12 | 0.47 |
| V_D_/V_T_ rest | 0.38 ± 0.13 | 0. 37 ± 0.13 | 0.38 ± 0.13 | 0.95 |
| PaO_2_ peak, mmHg | 79 ± 18 | 74 ± 22 | 79 ± 17 | 0.62 |
| PaCO_2_ peak, mmHg | 38 ± 5 | 38 ± 5 | 38 ± 5 | 0.65 |
| AaDO_2_ peak, mmHg | 35 (26) | 34 (14) | 36 (30) | 0.96 |
